# Supplementary material for: The incidence and mortality of childhood acute lymphoblastic leukemia in Indonesia: A systematic review and meta-analysis
Source: PLoS One. 2022 Jun 13;17(6):e0269706. doi: 10.1371/journal.pone.0269706 (PMC9191700; doi:10.1371/journal.pone.0269706)
Supplement: S1 Table — (DOCX) [file pone.0269706.s001.docx]

S1 Table. Medical subject heading (MeSH) terms used in each database

| Database | Medical subject heading | Number of studies found |
| --- | --- | --- |
| Pubmed | ("leukaemia"[All Fields] OR "leukemia"[MeSH Terms] OR "leukemia"[All Fields] OR "leukaemias"[All Fields] OR "leukemias"[All Fields] OR "leukemia s"[All Fields] OR ("acute lymphoblastic leukaemia"[All Fields] OR "precursor cell lymphoblastic leukemia lymphoma"[MeSH Terms] OR ("precursor"[All Fields] AND "cell"[All Fields] AND "lymphoblastic"[All Fields] AND "leukemia lymphoma"[All Fields]) OR "precursor cell lymphoblastic leukemia lymphoma"[All Fields] OR ("acute"[All Fields] AND "lymphoblastic"[All Fields] AND "leukemia"[All Fields]) OR "acute lymphoblastic leukemia"[All Fields]) OR ("precursor cell lymphoblastic leukemia lymphoma"[MeSH Terms] OR ("precursor"[All Fields] AND "cell"[All Fields] AND "lymphoblastic"[All Fields] AND "leukemia lymphoma"[All Fields]) OR "precursor cell lymphoblastic leukemia lymphoma"[All Fields] OR ("acute"[All Fields] AND "lymphoid"[All Fields] AND "leukemia"[All Fields]) OR "acute lymphoid leukemia"[All Fields]) OR ("acute lymphocytic leukaemia"[All Fields] OR "precursor cell lymphoblastic leukemia lymphoma"[MeSH Terms] OR ("precursor"[All Fields] AND "cell"[All Fields] AND "lymphoblastic"[All Fields] AND "leukemia lymphoma"[All Fields]) OR "precursor cell lymphoblastic leukemia lymphoma"[All Fields] OR ("acute"[All Fields] AND "lymphocytic"[All Fields] AND "leukemia"[All Fields]) OR "acute lymphocytic leukemia"[All Fields])) AND ("epidemiologies"[All Fields] OR "epidemiology"[MeSH Subheading] OR "epidemiology"[All Fields] OR "epidemiology"[MeSH Terms] OR "epidemiology s"[All Fields] OR ("epidemiology"[MeSH Subheading] OR "epidemiology"[All Fields] OR "prevalence"[All Fields] OR "prevalence"[MeSH Terms] OR "prevalance"[All Fields] OR "prevalences"[All Fields] OR "prevalence s"[All Fields] OR "prevalent"[All Fields] OR "prevalently"[All Fields] OR "prevalents"[All Fields]) OR ("epidemiology"[MeSH Subheading] OR "epidemiology"[All Fields] OR "incidence"[All Fields] OR "incidence"[MeSH Terms] OR "incidences"[All Fields] OR "incident"[All Fields] OR "incidents"[All Fields]) OR ("epidemiology"[MeSH Subheading] OR "epidemiology"[All Fields] OR "frequency"[All Fields] OR "epidemiology"[MeSH Terms] OR "frequence"[All Fields] OR "frequences"[All Fields] OR "frequencies"[All Fields])) AND ("indonesia"[MeSH Terms] OR "indonesia"[All Fields] OR "indonesia s"[All Fields] OR "indonesias"[All Fields] OR ("indonesian"[All Fields] OR "indonesians"[All Fields])) AND ("childhood"[All Fields] OR "childhoods"[All Fields] OR ("paediatrics"[All Fields] OR "pediatrics"[MeSH Terms] OR "pediatrics"[All Fields] OR "paediatric"[All Fields] OR "pediatric"[All Fields])) | 27 |
| Medline | ((("acute lymphoblastic leukaemia"[All Fields] OR "precursor cell lymphoblastic leukemia-lymphoma"[MeSH Terms] OR ("precursor"[All Fields] AND "cell"[All Fields] AND "lymphoblastic"[All Fields] AND "leukemia-lymphoma"[All Fields]) OR "precursor cell lymphoblastic leukemia-lymphoma"[All Fields] OR ("acute"[All Fields] AND "lymphoblastic"[All Fields] AND "leukemia"[All Fields]) OR "acute lymphoblastic leukemia"[All Fields]) OR ("leukaemia"[All Fields] OR "leukemia"[MeSH Terms] OR "leukemia"[All Fields])) OR ("acute lymphocytic leukaemia"[All Fields] OR "precursor cell lymphoblastic leukemia-lymphoma"[MeSH Terms] OR ("precursor"[All Fields] AND "cell"[All Fields] AND "lymphoblastic"[All Fields] AND "leukemia-lymphoma"[All Fields]) OR "precursor cell lymphoblastic leukemia-lymphoma"[All Fields] OR ("acute"[All Fields] AND "lymphocytic"[All Fields] AND "leukemia"[All Fields]) OR "acute lymphocytic leukemia"[All Fields])) OR ("precursor cell lymphoblastic leukemia-lymphoma"[MeSH Terms] OR ("precursor"[All Fields] AND "cell"[All Fields] AND "lymphoblastic"[All Fields] AND "leukemia-lymphoma"[All Fields]) OR "precursor cell lymphoblastic leukemia-lymphoma"[All Fields] OR ("acute"[All Fields] AND "lymphoid"[All Fields] AND "leukemia"[All Fields]) OR "acute lymphoid leukemia"[All Fields]) AND (("prevalence"[MeSH Terms] OR "epidemiology"[MeSH Terms]) OR "incidence"[MeSH Terms]) OR "epidemiology"[MeSH Terms] AND ("indonesia"[MeSH Terms] OR "indonesia"[All Fields]) OR ("indonesia"[MeSH Terms] OR "indonesia"[All Fields]) | 77 |
| Cochrane Library | (acute lymphoblastic leukemia):ti,ab,kw AND (Indonesia):ti,ab,kw | 5 |
| Google Scholar | allintitle: Indonesia Childhood OR Pediatric OR Indonesia OR Indonesian OR Incidence OR Epidemiology OR Frequency OR Prevalence "leukemia" | 63 |
| Science Direct | (Acute lymphoblastic leukemia) AND (Indonesia OR Indonesian) AND (Childhood OR Pediatric) | 49 |
| Indonesian Scientific Journal Database* | Acute lymphoblastic leukemia | 15 |
|  | Leukemia limfoblastik akut | 28 |
| Neliti* | Acute lymphoblastic leukemia | 9 |
|  | Leukemia limfoblastik akut | 19 |
| Indonesia One Search | Acute lymphoblastic leukemia AND Indonesia | 41 |
|  | Leukemia limfoblastik akut AND Indonesia | 51 |

*Database does not allow searches to be conducted using Boolean terms
