# Supplementary material for: The incidence and mortality of childhood acute lymphoblastic leukemia in Indonesia: A systematic review and meta-analysis
Source: PLoS One. 2022 Jun 13;17(6):e0269706. doi: 10.1371/journal.pone.0269706 (PMC9191700; doi:10.1371/journal.pone.0269706)
Supplement: S2 Table — (DOCX) [file pone.0269706.s002.docx]

S2 Table. Characteristics of included studies

| Author | Study Period (months) | Institution (City) | Grey literature | Number of acute lymphoblastic leukemia cases (male) | Number of mortality cases | Treatment protocol | Newcastle Ottawa scale | | | | |
| --- | --- | --- | --- | --- | --- | --- | --- | --- | --- | --- | --- |
|  |  |  |  |  |  |  | Selection | Comparability | Outcome | Total | Classification |
| Suryawan et al. (2007)^(35)^ | February - September 2016  (7) | Hasan Sadikin Hospital (Bandung) | No | 40 (30) | 10 | N/A | 4 | 1 | 1 | 6 | Moderate |
| Supriyadi et al. (2011)^(18)^ | January 1998 - December 2009 (143) | Dr. Sardjito Hospital (Special District of Yogyakarta) | No | 496 (297) | N/A | N/A | 4 | 2 | 3 | 9 | Good |
| Sutaryo et al. (2021)^(36)^ | June 2016 - November 2018 (29) | Dr. Sardjito Hospital (Special District of Yogyakarta) | Yes | 188 (114) | 30 | ALL 2016 | 4 | 2 | 3 | 9 | Good |
| Wijayanti & Supriyadi (2017)^(37)^ | January 2010-December 2015 (71) | Dr. Sardjito Hospital (Special District of Yogyakarta) | No | 309 (158) | N/A | N/A | 3 | 2 | 3 | 8 | Good |
| Simanjorang et al. (2013)^(38)^ | 1997-2008 (143) | Dharmais Hospital (Jakarta) | No | 72 (46) | 31 | N/A | 3 | 2 | 2 | 7 | Good |
| Yulianti & Adnan (2020)^(39)^ | 1 January 2013 - 31 December 2014 (24) | Harapan Kita Hospital (Jakarta) | No | 130 (81) | 10 | N/A | 3 | 2 | 2 | 7 | Good |
| Elisafitri et al. (2018)^(40)^ | 1 January 2014 - 31 December 2017 (47) | Dr. Wahidin Sudirohusodo Hospital (Makassar) | No | 109 (66) | 69 | N/A | 3 | 2 | 2 | 7 | Good |
| Larasati (2016)^(41)^ | January 2015 - November 2015 (10) | Soetomo Hospital (Surabaya) | Yes | 34 (22) | 16 | ALL 2013 | 4 | 2 | 1 | 7 | Good |
| Nency (2011)^(42)^ | July 2006 - December 2010 (53) | Dr. Kariadi Hospital (Semarang) | No | 119 (75) | N/A | ALL 2006 | 3 | 1 | 1 | 5 | Moderate |
| Samosir et al. (2021)^(43)^ | January 2014 - April 2019 (63) | Dr. Soetomo Hospital (Surabaya) | No | 49 (31) | N/A | ALL 2013 | 2 | 2 | 2 | 6 | Moderate |
| Meirizkia et al. (2021)^(44)^ | 2011-2018 (95) | Mohammad Hoesin Hospital (Palembang) | No | 230 (153) | 165 | ALL 2006 and ALL 2013 | 3 | 2 | 3 | 8 | Good |
| Perdani & Sudarmanto (2018)^(45)^ | May 2014 - May 2016 (144) | Dr. Kariadi Hospital (Semarang) | No | 55 (34) | N/A | ALL 2006 | 2 | 1 | 2 | 5 | Moderate |
| Widnyaya (2015)^(46)^ | January 2010 - December 2012 (35) | Sanglah Hospital (Denpasar) | Yes | 33 (18) | 23 | ALL 2006 | 2 | 2 | 2 | 6 | Moderate |

N/A, not available; ALL, acute lymphoblastic leukemia
