# Supplementary material for: The incidence and mortality of childhood acute lymphoblastic leukemia in Indonesia: A systematic review and meta-analysis
Source: PLoS One. 2022 Jun 13;17(6):e0269706. doi: 10.1371/journal.pone.0269706 (PMC9191700; doi:10.1371/journal.pone.0269706)
Supplement: S3 Table — (DOCX) [file pone.0269706.s003.docx]

S3 Table. Incidence of childhood acute lymphoblastic leukemia according to location, year in which the studies were conducted, and quality of studies

| Group | No. of studies | No. of patients | Incidence per 100,000 children (95% CI) | Prediction interval | Cochran Q; df | p-value |
| --- | --- | --- | --- | --- | --- | --- |
| **Location** | | | | | | |
| Java island | 11 | 1645 | 3.56 (1.99-5.13) | 1.67-7.58 | 5202.76; 10 | <0.001 |
| Non-Java islands | 2 | 219 | 8.51 (7.73-9.30) | 4.11-17.61 | 1483.81; 1 | <0.001 |
| **Study period** | | | | | | |
| <2010 | 3 | 687 | 5.12 (1.96-8.29) | 1.85-14.19 | 1536.63; 2 | <0.001 |
| ≥2010 | 10 | 1177 | 4.08 (2.07-6.09) | 1.84-9.06 | 4491.26; 9 | <0.001 |
| **Quality of study** | | | | | | |
| Good | 8 | 1568 | 4.80 (2.60-6.99) | 1.96-11.74 | 4556.57; 7 | <0.001 |
| Moderate | 5 | 296 | 3.56 (0.86-6.25) | 0.97-13.14 | 1814.80; 4 | <0.001 |
