# Supplementary material for: The incidence and mortality of childhood acute lymphoblastic leukemia in Indonesia: A systematic review and meta-analysis
Source: PLoS One. 2022 Jun 13;17(6):e0269706. doi: 10.1371/journal.pone.0269706 (PMC9191700; doi:10.1371/journal.pone.0269706)
Supplement: S1 Fig — (DOCX) [file pone.0269706.s004.docx]

**Identification of studies via other methods**

**Identification of studies via databases and registers**

Records identified from:

Citation searching (n = 12)

Hand searching (n = 15)

Records removed *before screening*:

Duplicate records removed (n =71)

Records identified from:

Medline (n=77)

Cochrane Library (n=5)

Pubmed (n=27)

Google Scholar (n=63)

Science Direct (n=49)

Indonesian Scientific Journal Database (n=43)

Neliti (n=28)

Indonesia One Search (n=92)

**Identification**

Records screened

(n = 313)

Records excluded via automation screening (n = 0)

Reports not retrieved after title and abstract screening (n = 10)

Reports sought for retrieval

(n = 27)

Reports sought for retrieval

(n = 313)

Reports not retrieved after title and abstract screening (n = 23)

**Screening**

Reports excluded:

- Not related to leukemia or mixed types of leukemia (n=81)
- Review studies (n=76)
- Studies not done in Indonesia (n=57)
- Animal studies (n=19)
- Not a cohort study (n=10)
- Population already included (n=14)
- Overlapping studies (n=20)
- Excluded after sensitivity analysis (n=3)

Reports assessed for eligibility

(n = 17)

Reports excluded:

- Studies not done in Indonesia (n=5)
- No full paper (n=9)

Reports assessed for eligibility

(n = 290)

Studies included in systematic review (n = 13)

Studies included in meta-analysis (n=13)

**Included**

S1 Fig. PRISMA flowchart for selection of included studies
