# Supplementary material for: The incidence and mortality of childhood acute lymphoblastic leukemia in Indonesia: A systematic review and meta-analysis
Source: PLoS One. 2022 Jun 13;17(6):e0269706. doi: 10.1371/journal.pone.0269706 (PMC9191700; doi:10.1371/journal.pone.0269706)
Supplement: S2 Fig — Galbraith plot (A), funnel plot (B), and trim-and-fill analysis funnel plot using R0 (C) of the incidence of childhood acute lymphoblastic leukemia. The observed effect size for the trim-and-fill analysis is 4.32 (95% CI 2.66–5.99), while the observed and imputed effect size is 3.00 (95% CI 1.07–4.92). (DOCX) [file pone.0269706.s005.docx]

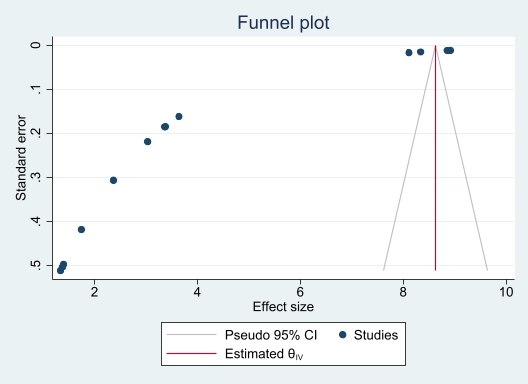

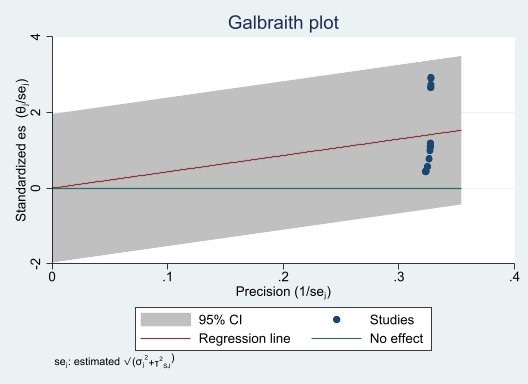


B

A


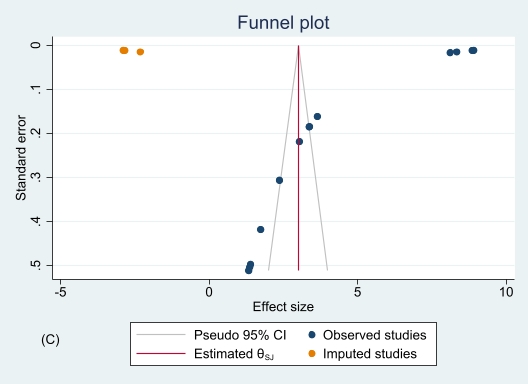


S2 Fig. Galbraith plot (A), funnel plot (B), and trim-and-fill analysis funnel plot using R_0_ (C) of the incidence of childhood acute lymphoblastic leukemia. The observed effect size for the trim-and-fill analysis is 4.32 (95% CI 2.66-5.99), while the observed and imputed effect size is 3.00 (95% CI 1.07-4.92).
