# Supplementary material for: The incidence and mortality of childhood acute lymphoblastic leukemia in Indonesia: A systematic review and meta-analysis
Source: PLoS One. 2022 Jun 13;17(6):e0269706. doi: 10.1371/journal.pone.0269706 (PMC9191700; doi:10.1371/journal.pone.0269706)
Supplement: S3 Fig — Galbraith plot of childhood acute lymphoblastic leukemia incidence in males (A) and females (B). (DOCX) [file pone.0269706.s006.docx]

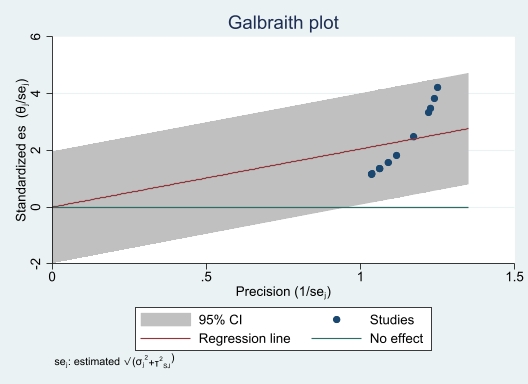

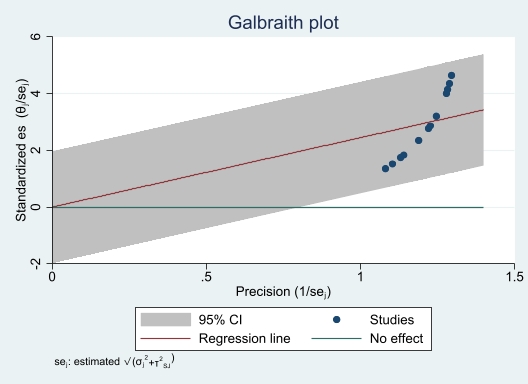
S3 Fig. Galbraith plot of childhood acute lymphoblastic leukemia incidence in males (A) and females (B).

B

A
