# Supplementary material for: The incidence and mortality of childhood acute lymphoblastic leukemia in Indonesia: A systematic review and meta-analysis
Source: PLoS One. 2022 Jun 13;17(6):e0269706. doi: 10.1371/journal.pone.0269706 (PMC9191700; doi:10.1371/journal.pone.0269706)
Supplement: S4 Fig — Funnel plot (A) and trim-and-fill analysis using R0 (B) of the incidence of childhood acute lymphoblastic leukemia in males. The observed effect size for the trim-and-fill analysis is 2.93 (95% CI 2.76–3.08), while the observed and imputed effect size is 3.05 (95% CI 2.90–3.19). (DOCX) [file pone.0269706.s007.docx]

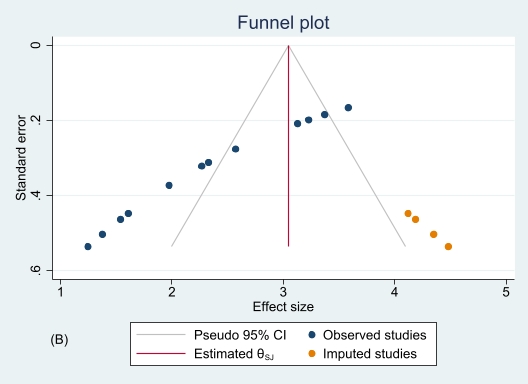

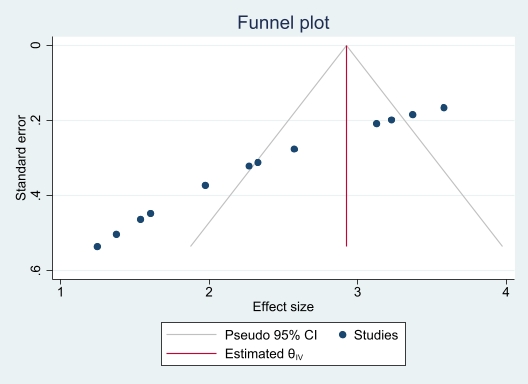


A

S4 Fig. Funnel plot (A) and trim-and-fill analysis using R_0_ (B) of the incidence of childhood acute lymphoblastic leukemia in males. The observed effect size for the trim-and-fill analysis is 2.93 (95% CI 2.76-3.08), while the observed and imputed effect size is 3.05 (95% CI 2.90-3.19).
