# Supplementary figures and images for: The incidence and mortality of childhood acute lymphoblastic leukemia in Indonesia: A systematic review and meta-analysis
Source: PLoS One. 2022 Jun 13;17(6):e0269706. doi: 10.1371/journal.pone.0269706 (PMC9191700; doi:10.1371/journal.pone.0269706)

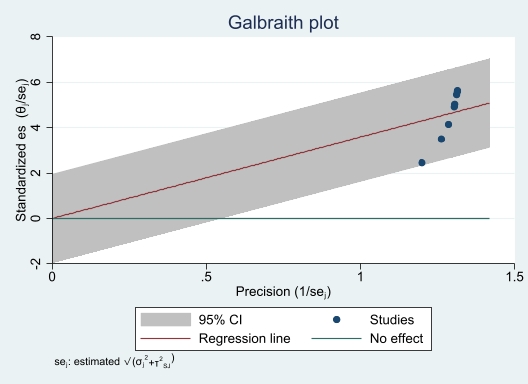


S6 Fig. Galbraith plot of the case fatality rate of childhood acute lymphoblastic leukemia.

Supplement: S6 Fig — (DOCX) [file pone.0269706.s009.docx]
